# Integrative Variation Analysis Reveals that a Complex Genotype May Specify Phenotype in Siblings with Syndromic Autism Spectrum Disorder

**DOI:** 10.1371/journal.pone.0170386

**Published:** 2017-01-24

**Authors:** Viviane Neri de Souza Reis, João Paulo Kitajima, Ana Carolina Tahira, Ana Cecília Feio-dos-Santos, Rodrigo Ambrósio Fock, Bianca Cristina Garcia Lisboa, Sérgio Nery Simões, Ana C. V. Krepischi, Carla Rosenberg, Naila Cristina Lourenço, Maria Rita Passos-Bueno, Helena Brentani

**Affiliations:** 1 LIM23-Institute of Psychiatry, University of São Paulo School of Medicine, São Paulo, Brazil; 2 Bioinformatics, Mendelics Análise Genômica, São Paulo, Brazil; 3 Department of Morphology and Genetics, Federal University of São Paulo, São Paulo, Brazil; 4 Department of Informatics, Federal Institute of Espírito Santo, Serra, Brazil; 5 Department of Genetics and Evolutionary Biology, Institute of Biosciences, University of Sao Paulo, São Paulo, Brazil; Yeshiva University Albert Einstein College of Medicine, UNITED STATES

## Abstract

It has been proposed that copy number variations (CNVs) are associated with increased risk of autism spectrum disorder (ASD) and, in conjunction with other genetic changes, contribute to the heterogeneity of ASD phenotypes. Array comparative genomic hybridization (aCGH) and exome sequencing, together with systems genetics and network analyses, are being used as tools for the study of complex disorders of unknown etiology, especially those characterized by significant genetic and phenotypic heterogeneity. Therefore, to characterize the complex genotype-phenotype relationship, we performed aCGH and sequenced the exomes of two affected siblings with ASD symptoms, dysmorphic features, and intellectual disability, searching for *de novo* CNVs, as well as for *de novo* and rare inherited point variations—single nucleotide variants (SNVs) or small insertions and deletions (indels)—with probable functional impacts. With aCGH, we identified, in both siblings, a duplication in the 4p16.3 region and a deletion at 8p23.3, inherited by a paternal balanced translocation, t(4, 8) (p16; p23). Exome variant analysis found a total of 316 variants, of which 102 were shared by both siblings, 128 were in the male sibling exome data, and 86 were in the female exome data. Our integrative network analysis showed that the siblings’ shared translocation could explain their similar syndromic phenotype, including overgrowth, macrocephaly, and intellectual disability. However, exome data aggregate genes to those already connected from their translocation, which are important to the robustness of the network and contribute to the understanding of the broader spectrum of psychiatric symptoms. This study shows the importance of using an integrative approach to explore genotype-phenotype variability.

## Introduction

Autism spectrum disorder (ASD) is a neurodevelopmental disorder [1] characterized by early-onset social and communication impairment, as well as restrictive stereotypic movements and behaviors [2]. Approximately 70% of ASD patients have at least one clinical or psychiatric comorbidity, whereas 48% have two or more [3]. In addition to presenting a heterogeneous phenotype, ASD is multifactorial disorder with a complex genetic architecture [4–7]. Various reviews have provided in-depth analyses of the degree of genetic and phenotypic association between ASD and genetic syndromes [8–12]. Miles et al. [13] defined complex ASD as that observed in a subset of individuals with evidence of abnormality occurring at the beginning of morphogenesis, manifesting as dysmorphic abnormalities but not associated with a known genetic syndrome. Therefore, syndromic ASD is represented by complex cases with or without an association with a known genetic syndrome.

Although common variations (with a minor allele frequency > 1%) are the largest genetic component of ASD, accounting for 40% of the risk [14,15], their individual effect is small [16]. Whole-genome screening for *de novo* and inherited copy number variations (CNVs) have revealed that rare variants are more common in ASD probands than in healthy controls and unaffected family members [17–22]. Comparisons of syndromic and non-syndromic cases of ASD have revealed that clinically relevant CNVs are more common in syndromic probands [23,24]. The pathogenicity of *de novo* CNVs has been related to CNV length, larger CNVs having been shown to have a greater impact [17,25–27]. However, recurrent CNVs have been associated with an increased risk of other neurodevelopmental disorders, such as schizophrenia and epilepsy [28]. Various authors have attempted to improve the understanding of the nature of CNVs and to elucidate their contribution to specific phenotypes [29,30]. In patients with developmental disorders, Andrews et al. [30] found that, if protein-protein interactions are taken into account, patients with genic (especially *de novo*) CNVs, disrupting the same cluster of functionally related genes, present phenotypes that are more similar than would be expected. Whole-exome sequencing studies have reported that *de novo* non-synonymous single nucleotide variants (SNVs) and small insertions and deletions (indels) are strongly associated with ASD [31–34]. Iossifov et al. [35] showed that almost all gene-disrupting mutations occur opposite wild-type alleles, that 13% of *de novo* missense mutations account for 12% of diagnoses, and that 43% of likely gene-disrupting *de novo* mutations account for 9% of diagnoses. A whole-genome sequencing study of 85 quartet families (two parents and two ASD-affected siblings) found that 69.4% of the affected siblings carried different ASD-relevant mutations (*de novo* and rare inherited SNVs or structural variants) and that sibling pairs with discordant mutations usually showed more clinical variability than did those who shared a risk variant [36].

The prevalence of CNVs suggests that chromosomal microarrays should be used in the genetic analysis of syndromic ASD or familial cases of the disorder, especially when the patient has an intellectual disorder [37]. Patients with localized or generalized syndromic overgrowth should also be tested, given that the condition can be caused by subtle genomic rearrangements [38]. It is of note that even in the presence of large pathogenic CNVs, increasing heterogeneity and complexity of a phenotype usually imply a greater number of variants. Assuming that different ASD phenotypes could be the product of multiple biological pathways, possibly affecting the same brain mechanisms, more studies should focus on systems genetics and network analyses [39,40]. This underscores the importance of gaining a better understanding of the role that sets of variant genes play in phenotype differences, which could be achieved through comparative studies of affected individuals with shared and distinct phenotypic effects of rare alleles [40]. Therefore, to characterize the complex genotype-phenotype relationship, we performed an analysis of CNVs and rare point variations by conducting array comparative genomic hybridization (aCGH) and exome sequencing in two syndromic siblings with intellectual disability (ID) who shared various dysmorphic features, although they showed significant clinical differences in terms of the psychiatric phenotype.

## Materials and Methods

### Clinical assessment of the subjects

Two siblings (one male and one female) with syndromic ASD and their parents volunteered to participate in an ongoing exome research study at the Autism Spectrum Disorder Program Clinic of the Psychiatric Institute of the University of São Paulo School of Medicine. The study was approved by the Research Ethics Committee for the Analysis of Research Projects of the University of São Paulo School of Medicine *Hospital das Clínicas* (Ruling no. 0692–11). All subjects gave written informed consent. The siblings underwent evaluation by specialized clinicians. The diagnosis of ASD was based on the criteria established in the Diagnostic and Statistical Manual of Mental Disorders, fifth edition, as well as on the results of morphological and neurological examinations. The Childhood Autism Rating Scale (CARS) was used in order to determine the severity of the ASD symptoms. The results of karyotyping, screening for innate metabolism errors (testing for amino acids and saccharides in urine and serum), and the test for fragile X syndrome were all negative.

### CNV analysis

We performed aCGH on a commercial whole-genome 180K platform containing approximately 180,000 oligonucleotide probes distributed throughout the human genome (Agilent Technologies, Santa Clara, CA, USA). A pool of healthy human DNA (Promega, Madison, WI, USA) was used as a reference in the experiments, and the procedure was performed as recommended by the manufacturer. Scanned images were processed using Feature Extraction 10.7.3.1 software (Agilent Technologies). We called copy number alterations on the basis of the log_2_ ratio of the Cy3/Cy5 intensities in a given genomic segment, > 0.3 or < −0.3 for gains or losses, respectively, using the aberration detection statistical algorithm aberration detection method 2, with a sensitivity threshold of 6.7. Because we were interested in pathogenic CNVs, we selected only *de novo* genic CNVs that were larger than 100 kb and were considered uncertain, likely pathogenic, or pathogenic according to ClinVar similarity regarding the CNV region (NCBI Variant Viewer, https://www.ncbi.nlm.nih.gov/variation/view/).

Imbalances identified with aCGH were validated by multiple ligation probe amplification (MLPA) with two probe sets (SALSA MLPA P036-E1 human telomere-3 and SALSA MLPA P070-B2 human telomere-5; MRC-Holland, Amsterdam, the Netherlands). Fluorescence *in situ* hybridization (FISH) was also use in order to determine whether the rearrangement had been inherited as a balanced translocation. The FISH was performed according to standard protocols.

### Exome sequencing

We extracted DNA from whole blood and quantified it with a fluorometer (Qubit; Life Technologies Corporation, Carlsbad, CA, USA). The exome was captured and enriched from genomic DNA using an exome enrichment kit (Nextera; Illumina Inc., San Diego, CA, USA), representing 62 Mbp of the human genome (hg19 build). Sequencing was performed with a high-resolution optical imaging system (HiScan; Illumina Inc.).

Fragments were aligned to the hg19 reference genome through the use of Burrows-Wheeler alignment [41], and variant calling was performed with a software package for the analysis of sequence data (Genome Analysis Toolkit; Broad Institute, Cambridge, MA, USA) [42–44]. A total of 800 informative single nucleotide polymorphisms were analyzed with the whole-genome association analysis toolset (PLINK; http://pngu.mgh.harvard.edu/~purcell/plink/) to confirm the familial relationship between samples [45]. For genotype calls (in all members of the quartet, we used only reads with a Phred score ≥ 30 [46], bases with ≥ 20 reads, and a genotype quality score ≥ 20 [33].

### SNV analysis

Variant annotation was performed with an annotation program (ANNOVAR, http://www.openbioinformatics.org/annovar/) [47]. To select rare variants (minor allele frequency ≤ 0.01) and novel variants, we employed the following databases: 1000 Genomes Project (http://www.1000genomes.org/); the National Heart, Lung, and Blood Institute GO Exome Sequencing Project (6500 exomes; http://evs.gs.washington.edu/EVS/); and the Exome Aggregation Consortium (http://exac.broadinstitute.org/). Variants with a depth of coverage of less than 20× reads in all members of the family were excluded in order to avoid false positives. To identify *de novo* variations, we accepted candidates only at loci that were homozygous for the reference allele in the parents. For the gene analysis, we selected only nonsynonymous SNVs, small indels, and splicing site variations. If present, we selected the variations that were considered conserved (score ≥ 2) in the Genomic Evolutionary Rate Profiling++ algorithm [48] were selected, as were those that were considered deleterious or possibly deleterious in the Polymorphism Phenotyping v2 HDIV algorithm [49].

### Integration of CNVs and exome variants: network analysis

We performed enrichment analyses with a web-based gene set analysis toolkit (WebGestalt; http://bioinfo.vanderbilt.edu/webgestalt/) [50], using a list of the brain-expressed genes among the CNVs. For those analyses, we considered only genes expressed in at least one sample from any brain area with positive expression according to the BrainSpan Atlas of the Developing Human Brain [51].

For the integrative approach, network analysis was performed with the Search Tool for the Retrieval of Interacting Genes/Proteins (STRING; http://string-db.org/), the minimum interaction confidence score being 0.4 (medium confidence). The STRING database comprises known, predicted protein interactions, as well as direct (physical) and indirect (functional) associations, derived from genomic context, high-throughput experiments, conserved coexpression, and text mining [52]. For prioritization, we used measures of centrality, such as degree, bridgeness, and brokering, Degree is the number of edges of a vertex (node), and vertices with a very high degree are known as hubs [53,54]. Bridgeness is a measure of the extent to which a node or edge is located between connected regions [55], and brokering is when a broker gene acts as a hub that connects many genes that do not otherwise connect with each other [56].

## Results

### Phenotypic similarities and differences between the siblings

The two patients evaluated were siblings born to a nonconsanguineous couple. There was no family history of ASD, other psychiatric disorders, ID, or malformations.

The male sibling had his first physician visit at 11 years of age (in 1992). At the time, the mother complained that the child had spoken individual words only after 2 years of age and had stopped producing any words at all by 4 years of age. He had difficulty learning new things at school, had no social interests, and was very attached to routines. The mother also stated that she had had an uneventful pregnancy and a vaginal delivery without complications. She could not remember the birth weight or length. However, she was able to remember that he had taken his first steps at 2 years of age. He was diagnosed with ID in 1992 and with ASD in 2003. He began to show irritability, mainly in the morning, and sometimes became autoaggressive or exhibited outwardly directed aggressive behavior. Since his first physician visit (in 1992), he had been under pharmacological treatment with antipsychotic agents.

The female sibling had her first physician visit at 7 years of age (also in 1992). At the time, the mother complained that the girl had difficulties learning new things at school and that her social skills were extremely inadequate. She had taken her first steps at 18 months of age, and she had not begun to produce speech until after 2 years of age. The mother reported having had abdominal pain and vaginal bleeding during pregnancy and that it had been a cesarean delivery (birth weight: 4140 g; birth length: 51 cm). The patient was diagnosed with ID in 1992 and with ASD in 2003. Since her first physician visit, she exhibited psychomotor agitation, mainly in the mornings. She was occasionally aggressive, although she was an affectionate and gentle child most of the time. She liked playing with other children. However, she sometimes exhibited periods of inappropriate, hypersocial behavior and periods during which she could not stop talking and presented sleep difficulties. Since 1993 (when she was 8 years of age), she had been under pharmacological treatment with antipsychotic agents.

In the most recent medical evaluation (S1 Table), the male sibling met the criteria for a diagnosis of ASD according to the Diagnostic and Statistical Manual of Mental Disorders, fifth edition [2], whereas the female sibling was diagnosed with ID and syndromic features. The CARS interview with the parents resulted in a score of 33.5 for the male sibling and 24.0 for the female sibling (details of the psychiatric examination and neurological evaluation of the siblings are provided in S1 Table).

### aCGH and MLPA analyses

**Fig 1:**
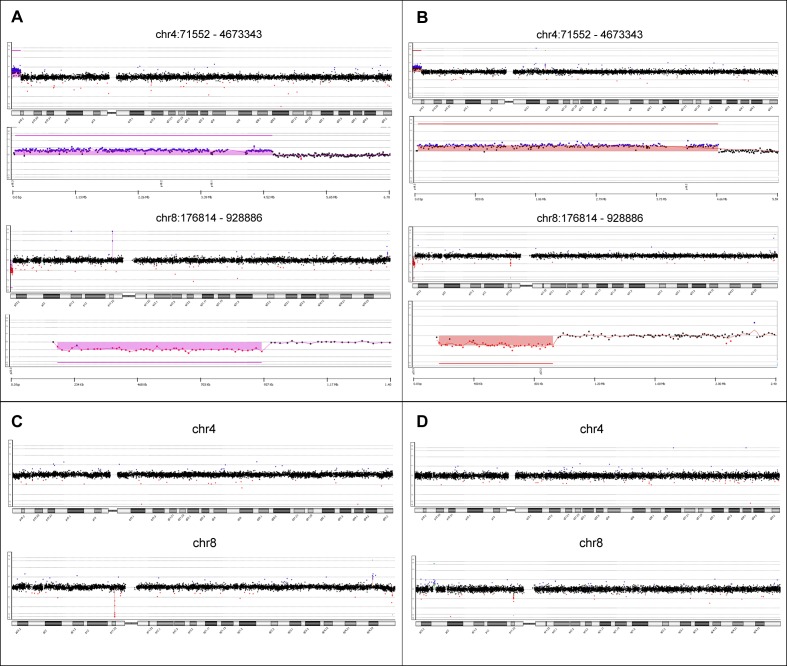
Chromosomal alterations detected with aCGH analysis in both siblings. A and B present the aCGH profiles of chromosomes 4 and 8, showing the 4p16.3 duplication and the 8p23.3 deletion in the male and female siblings, respectively; C and D present the normal copy number profiles of chromosomes 4 and 8 of the father and mother, respectively.

The syndromic presentation suggested the existence of a chromosomal alteration, which was investigated with aCGH (Fig 1). In the two siblings, there were 12 *de novo* CNVs (S2 Table): 8 in the female and 4 in the male. Filtering revealed that both siblings had 2 terminal chromosomal alterations: a 4.6-Mb duplication at 4p16.2-p16.3 (chr4:71552–4673343; hg19); and a 752-kb deletion at 8p23.3 (chr8:176,814–928,886; hg19). The MLPA analysis confirmed the presence of the 4p16 duplication and the 8p23 deletion (S1 Fig).

### FISH analysis

**Fig 2:**
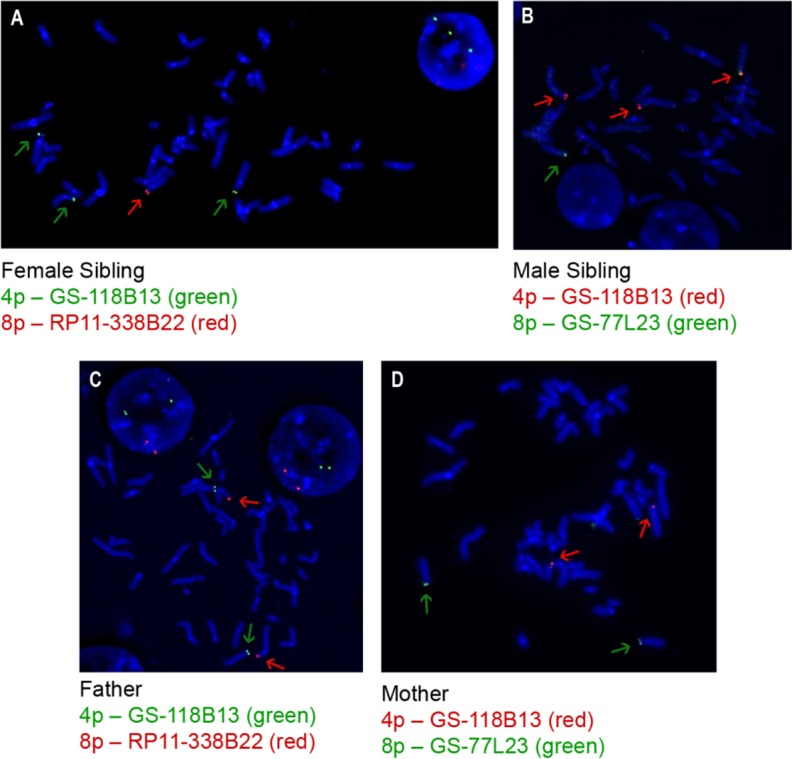
FISH images. (A) image showing that, in the female sibling, there were three chr4p copies, marked in green with a GS-118B13 probe (green arrow), and one chr8p copy, marked in red with a RP11-338B22 probe (red arrow); (B) image showing that, in the male sibling, there were also three chr4p copies, marked in red with a GS-118B13 probe (red arrow), and one chr8p copy, marked in green with a GS-77L23 probe (green arrow); (C) image showing that, in the father, there was a balanced translocation, t(4;8); and (D) image showing that, in the mother, the chromosomes were normal.

The FISH analysis confirmed the copy number alterations detected in the siblings (4p16.3 duplication and 8p23.1 deletion, as shown in Fig 2A and Fig 2B, respectively), and the chromosomal imbalances were derived from a translocation between chromosomes 4 and 8 [der(8)t(4;8)(p16.3;p23.1)]. The FISH analysis performed in the parents revealed that the father (Fig 2C) was a carrier of the balanced translocation t(4;8)(p16.3;p23.1).

### Phenotype and chromosomal alterations correlation

Various cases of translocations between 4p16.6 and 8p23.1 have been described, the first having been reported by Goggin et al. [57]. Table 1 summarizes the characteristics observed in our patients compared with those of other reported cases, indicating what is typically found in cases of pure 4p duplication and pure 8p deletion [57–66].

**Table 1:** Summary of the characteristics observed in our patients, compared with those of other cases described in the literature.

Characteristic	Sibling 1	Sibling 2	Skrlec et al. [58]	Mau et al. [59]	Partington et al. [60]		Goggin et al. [57]	Literature on dup(4p)*	Literature on del(8p)*
**Sex**	Male	Female	Male	Female	Female (1)	Female (2)	Male	NA	NA
**Age at evaluation**	33 years	29 years	5 months	2 years, 10 months	15 years	16 years	NA	NA	NA
**Cytogenetic alterations**	46,XY, der(8)t(4;8) (p16.3;p23.1)	46,XX, der(8)t(4;8) (p16.3;p23.1)	46,XY,der(8)t(4;8)(p16.1;p23.1).ish der(8)t(4;8) (D8-S504-,WHCR+, D8Z2+)dn	46,XX,der(8) t(4;8)(p12;p23.1)	46,XX,der(8) t(4;8)(p16.2; p23.1)	46,XX,der(8) t(4;8)(p16.2; p23.1)	46,XY,der(8) t(4;8)(p12; p23)	NA	NA
**Neonatal period**									
**Birth height (cm)**			52	44				NA	NA
**Birth weight (g)**			3360	2450				NA	NA
**Birth OFC (cm)**			35	31				NA	NA
**Anthropometric**									
**Height (cm)**	190	183		84.5				NA	NA
**Weight (kg)**	95.8	91.5		11,2				NA	NA
**OFC (cm)**	60	59		46,5				NA	NA
**Arm span (cm)**	194	185		NA				NA	NA
**Dysmorphic features**									
**Microcephaly**	-	-		+			+	+	+
**Macrocephaly**	+	+				+			
**Round face**	-	-		+					
**Long face**	+	+							
**Short neck**	-	-					+	+	+
**Low anterior hairline**	-	-		+					
**Low posterior hairline**	+	+		+				+	
**Arched eyebrows**	+	+		+				+	
**Long eyelashes**	+	+		+				+	
**Synophrys**	-	-		+				+	
**Hirsutism**	-	-	+	+				+	
**Microphthalmia**	-	-						+	+
**Epicanthic folds**	-	-							+
**Hypertelorism**	-	-						+	+
**Wide nasal bridge**	+	+	+	+			+	+	+
**Anteverted nares**	-	-	+	+					
**Long philtrum**	-	-		+				+	
**Thin lips**	-	+		+					+
**Down-turned corners of the mouth**	-	-		+					
**Micrognathia or Retrognathia**	-	-	+					+	+
**Prognathism**	-	-							
**Widely spaced teeth**	-	-		+				+	
**Crowded teeth**	+	+							
**Low-set ears**	+	+	+	+				+	+
**Malformed ears**	+	+		+				+	+
**Widely spaced nipples**	+	NA						+	+
**Large hands**	+	**+**			+	+			
**Short tapering fingers**	-	**-**						+	
**Proximal implantation of thumbs**	-	**-**		+					+
**Arachnodactyly**	-	**-**						+	
**Clinodactyly**	+	**+**	+					+	
**Large feet**	+	**+**			+	+			
**Proximal implantation of toes**	-	**-**	+						
**Syndactyly**	-	**-**						+	
**Transverse palmar crease**	-	**-**		+				+	
**Hypoplastic nails**	-	**+**		+					+
**Rectus abdominis diastasis**	-	**-**	+						
**Cryptorchidism**	-	**-**	+					+	
**Hypospadias**	-	**-**						+	+
**Malformative features**									
**Cardiopathy**			AVSD					+	+
**Genitourinary anomalies**			Vesicoureteral reflux	Small clitoris				+	+
**Hypoplasia of the corpus callosum**			+						
**Coloboma of the iris or retina**	-	**-**					Both		
**Other**									
**Behavior features**									
**Intellectual disability**	+	+	+	+	+	+	+		+
**Seizures**	-	-		-				+	+
**Stereotypic movements**	+	-		+					
**Hyperactive**	-	-		+					+
**Obsessional behavior**	-	-			+				
**Feeding difficulties**	-	-		+				+	+
**Recurrent infections**	-	-		+					
**Other**	-	-		NHS					

NA: not applicable; OFC: occipitofrontal circumference; AVSD: atrioventricular septal defect; NHS: neonatal hypocalcemic seizure.

*[61–66]

### Genes in chromosomal alterations

To explore the phenotypes associated with the chromosomal alterations detected, we conducted an enrichment analysis based on the 69 genes present in the 4p duplication, expressed in brain tissue and present in the WebGestalt database (of the 80 RefSeq genes present in the region according to the hg19 build from the UCSC Genome Browser). The disease enrichment analysis (S3 Table) revealed that 5 genes *(WHSC1*, *FGFR3*, *SH3BP2*, *LETM1*, and *STX18*) were found to be overrepresented in craniofacial abnormalities (adjusted *p*-value = 0.0002), musculoskeletal abnormalities (adjusted *p*-value = 0.0009) and congenital abnormalities (adjusted p-value = 0.0040). Other genes, such as *GAK*, *HTT*, *TMEM175*, *FAM193A*, and *DGKQ*, were mainly related to movement disorders (adjusted *p*-value = 0.0001) and basal ganglia disease (adjusted *p*-value = 3.73e-05). The gene ontology analysis revealed enrichment of fibroblast growth factor-activated receptor activity (*FGFR3* and *FGFRL1* genes, adjusted *p*-value = 0.0082) and rough endoplasmic reticulum (*NAT8L*, *LRPAP1*, and *HGFAC* genes, adjusted *p*-value = 0.0445).

The 8p23.1 deletion affects 6 brain-expressed genes (from a total of 8 RefSeq genes according to the hg19 build from the UCSC Genome Browser): *RPL23AP53*, *ZNF596*, *FAM87A*, *FBXO25*, *TDRP*, and *ERICH1*. As expected, there was no significant functional enrichment.

Using the STRING, we searched possible connections described in the literature and protein-protein interaction databases connecting all the CNV genes. There were 61 genes represented in the STRING (S2 Fig), with 32 connected proteins (i.e., 52.45%, 32/61) and 29 interactions, representing enrichment of interactions (*p*-value = 3.09e-14). In addition, genes overrepresented in craniofacial abnormalities, musculoskeletal abnormalities, syndromic abnormalities, or congenital abnormalities were all presented in the biggest component of the network formed (the component with the most number of connecting genes). The genes *ZNF596* and *FBXO25* from 8p23.1 appeared to be connected, although not within the biggest component of the network (S2 Fig).

### Exome sequencing

We sequenced 45 million reads per sample (4.5 Gb). To avoid miscalling variants [67], we removed polymerase chain reaction duplicates, resulting in 32 million reads (raw data submitted at the European Nucleotide Archive, accession number PRJEB15585). The mean coverage on target was 35×, and 44.63% of the targets showed at least 20× coverage (S4 Table).

After the SNV filtering process, only 1 *de novo* variant was identified, disrupting the *TUBAL3* gene in the exome of the female sibling. *TUBAL3* encodes the tubulin alpha chain-like 3 protein and occurs in exon 4, promoting the replacement of glutamate with lysine at position 174 and considered deleterious according to the Polymorphism Phenotyping v2 and Genomic Evolutionary Rate Profiling++ algorithms. In the male sibling data, we identified 3 rare X-chromosome variants (*ADGRG2*, *CNGA2*, and *FIGF*), as can be seen in Table 2.

**Table 2:** Details of the hemizygous variations found in the exome of the male sibling.

Chr	Start	End	Ref	Alt	Gene	Variation	1000G	ESP6500	ExAC	dbSNP ID	Polyphen2	GERP++
X	19032163	19032163	G	T	*ADGRG2*	Nonsyn. SNV	0.0013	0.0017	0.0006	rs150013899	0.745	3.37
X	15376167	15376167	G	C	*FIGF*	Nonsyn. SNV	NA	NA	NA	NA	0.804	3.30
X	150906999	150906999	A	C	*CNGA2*	Nonsyn. SNV	0.0079	0.0085	0.0025	rs142562634	0.981	3.69

Chr: chromosome; Ref: reference allele; Alt: altered allele; 1000G: 1000 Genomes Project (database); ESP6500: National Heart, Lung, and Blood Institute 6500 exome GO Exome Sequencing Project (database); ExAC: Exome Aggregation Consortium (database); dbSNP: Single Nucleotide Polymorphism Database; Polyphen2: Polymorphism Phenotyping v2 (algorithm); GERP++: Genomic Evolutionary Rate Profiling++ (algorithm); Nonsyn.: nonsynonymous; SNV: single nucleotide variant; NA: not applicable.

**Fig 3:**
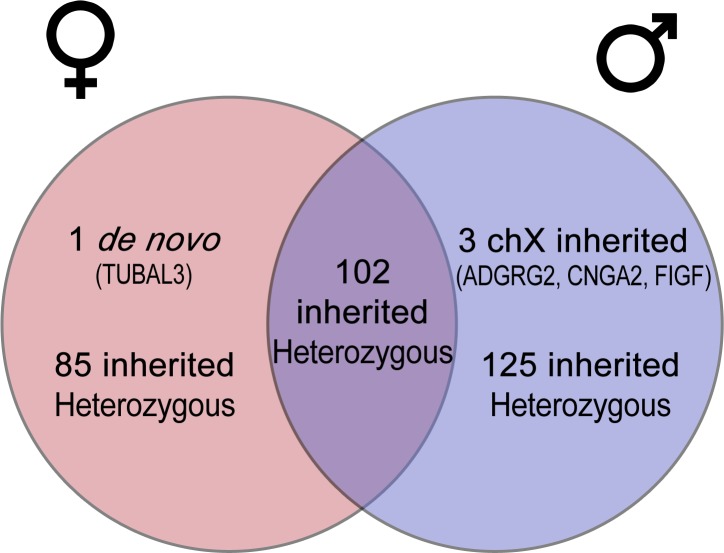
Summary diagram of the variations found in the two siblings.

As for the deleterious heterozygous variants inherited from one of the parents (Fig 3), we identified a total of 312 variants (S5 Table): in the male sibling, there were 125 exclusive variants (109 non-synonymous, 8 small indels, 5 stop-gains, 1 stop-loss, and 2 splicing sites); in the female sibling, there were 85 exclusive variants (81 nonsynonymous SNVs, 2 small indels, and 2 stop-gains); and the siblings shared 102 variants (96 nonsynonymous SNVs, 5 frameshift indels, and 1 non-frameshift indel).

### Integrative network analysis of CNVs and exome variants

**Fig 4:**
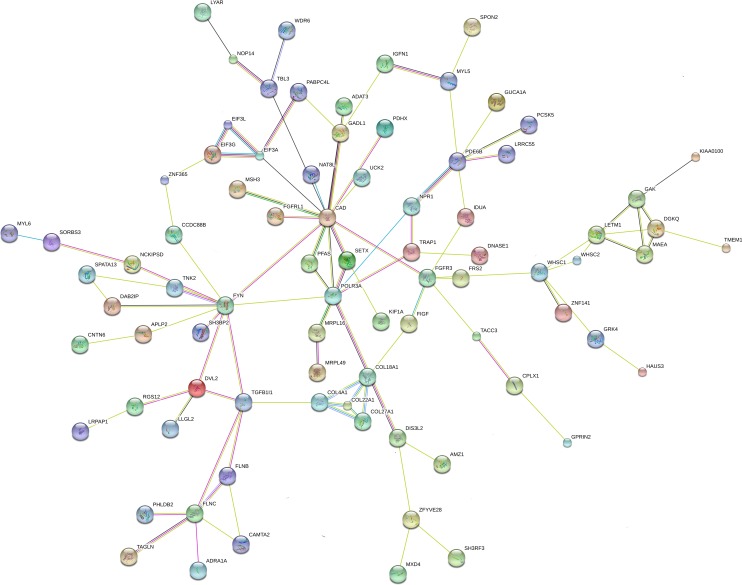
Male sibling STRING network analysis. Biggest component of the connecting brain-expressed genes present in the unbalanced translocation, altered by the shared and exclusive rare inherited SNV and indels found in the male sibling.

**Fig 5:**
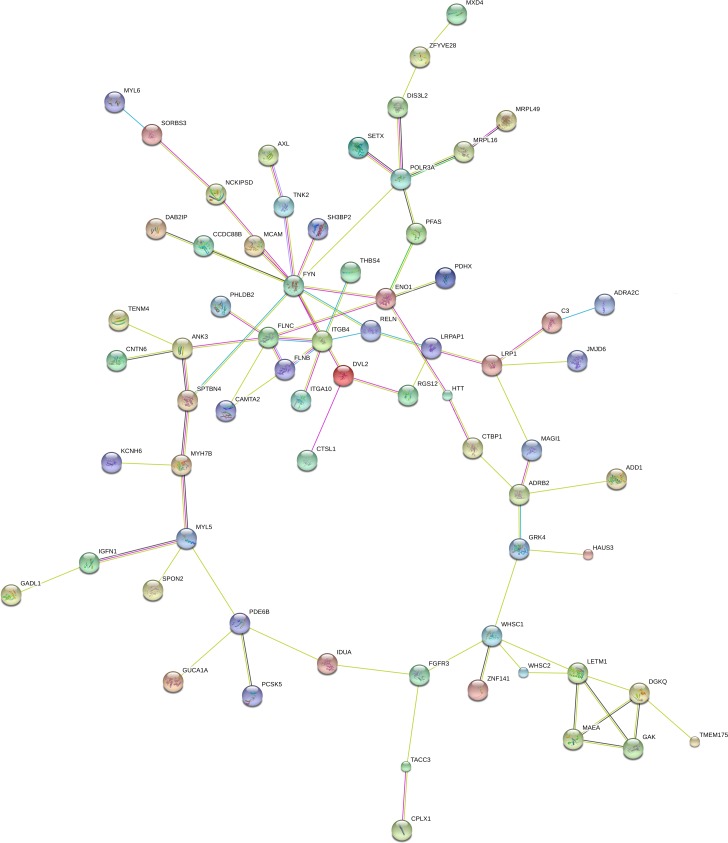
Female sibling STRING network analysis. Biggest component of the connecting brain-expressed genes present in the unbalanced translocation, altered by the shared and exclusive rare inherited SNV and indels found in the female sibling.

Using the combined brain-expressed gene list from the male sibling (70 genes present in the CNVs, 81 genes with variants shared by both siblings, and 105 genes with exclusive variants), we searched all connections using STRING and found enrichment of interactions (number of nodes: 236; number of edges: 129; average node degree: 1.09; clustering coefficient: 0.811; expected number of edges: 86 and protein-protein interaction enrichment *p*-value: 1.19e-05). Using the combined brain-expressed gene list from the female sibling (70 genes present in the CNVs, 81 genes with variants shared by both sibling; and 71 genes with exclusive variants), we searched all connections with STRING and again found enrichment of interactions (number of nodes: 204; number of edges: 101; average node degree: 0.99; clustering coefficient: 0.812; expected number of edges: 58; and protein-protein interaction enrichment p-value: 1.89e-07). Figs 4 and 5 show the biggest connected component for the male sibling and female sibling, respectively.

We selected genes connected in the biggest component of each sibling network analysis: 82 connecting genes for the male sibling (26 located in the 4p duplication) and 68 connecting genes for the female sibling (26 in the 4p duplication). The biggest connecting component in the networks of both siblings shared 46 genes. Therefore, 36 genes appeared to be connected exclusively in the network of the male, compared with 22 in the network of the female. After calculating the centrality measures for each network (S6 Table for the male and S7 Table for the female), we identified the broker and bridge genes (5% higher brokering score for the brokers, and 5% higher bridging scores for the bridges), as detailed in Table 3. There were 5 broker genes in the male (*CAD*, *FGFR3*, *PDE6B*, and *FYN*) and 3 in the female (*FLNC*, *ITGB4*, and *FYN*). There were 3 bridge genes in the biggest connected component of the network of the female: *CTBP1*, *HTT* (present in the translocation but connected only in the female network), and *SPTBN4* (exclusive, i.e. altered only in the female). *IDUA*, *NCKIPSD*, *FIGF*, and *NPR1* are bridge genes in the network of the male, the last 2 being exclusive. The *de novo* variant *TUBAL3* did not appear to be connected in the network of the female. Only one hemizygous variant gene (the bridge gene *FIGF*) was connected in the network of the male.

**Table 3:** Comparison of broker and bridge genes in the biggest connected components of the networks of the two siblings.

Sib	Gene	Betweenness	Bridgeness	Brokering	Closeness	Clustering	Degree	Bridge or broker?*	Variation type	Exclusive?^†^
M	*CAD*	1816.83	15.939	0.167	0.379	0.033	14	Broker	Nonsyn. SNV	Yes
M	*FGFR3*	1232.00	74.089	0.074	0.333	0.000	6	Broker	4p Dup	No
M	*FYN*	1471.83	35.496	0.118	0.355	0.044	10	Broker	Nonsyn. SNV	No
M	*PDE6B*	346.67	13.867	0.074	0.250	0.000	6	Broker	4p Dup	No
M	*POLR3A*	907.83	45.305	0.085	0.345	0.143	8	Broker	Nonsyn. SNV	No
F	*FLNC*	224.52	15.273	0.078	0.236	0.133	6	Broker	Nonsyn. SNV	No
F	*FYN*	1155.52	14.664	0.176	0.288	0.015	12	Broker	Nonsyn. SNV	No
F	*ITGB4*	224.47	12.827	0.078	0.242	0.133	6	Broker	Nonsyn. SNV	Yes
M	FIGF**	133.00	159.600	0.025	0.263	0.000	2	Bridge	Nonsyn. SNV	Yes
M	*IDUA*	159.50	239.250	0.025	0.265	0.000	2	Bridge	4p Dup	No
M	*NCKIPSD*	158.00	131.667	0.025	0.266	0.000	2	Bridge	Nonsyn. SNV	No
M	*NPR1*	227.17	121.156	0.025	0.272	0.333	3	Bridge	Nonsyn. SNV	Yes
F	*CTBP1*	396.83	264.556	0.030	0.223	0.000	2	Bridge	4p Dup	Yes
F	*HTT*	416.33	297.381	0.030	0.239	0.000	2	Bridge	4p Dup	Yes
F	*SPTBN4*	567.50	283.750	0.045	0.262	0.000	3	Bridge	Nonsyn. SNV	Yes

Sib: Sibling; M: Male; F: Female; Nonsyn.: nonsynonymous; Dup: duplication.

*bridging and brokering scores: 95th percentile.

^†^Connected in biggest network of only one sibling.

**Hemizygous variation.

## Discussion

Our major goal in this paper was to assess genetic findings in siblings that shared syndromic features and ID, a secondary goal being to explain the differences in the presentation of their psychiatric symptoms. According to Miles et al. [13,68], the male sibling could be classified as having complex autism (autism with ID and syndromic features). Both siblings presented irritability, agitation, and aggressive behavior, symptoms that are present in cases of ASD, as well as in those of ID only. Even among individuals with ASD, these symptoms are more common in those with ID [69].The male sibling had an attachment to repetitive behaviors, did not make eye contact, showed no capacity for joint attention, had no social interests, and had no communicative ability, all of which were symptoms that were not present in the female sibling. Their social impairments and alterations in social skills differed, and the female sibling had been described as presenting periods of inappropriate, hypersocial behavior and periods during which she could not stop talking and had sleep difficulties.

The duplication at 4p16.3 and deletion at 8p23.1, both of which were found in both siblings, are located in a recurrent translocation region in olfactory receptor gene clusters commonly correlated with non-allelic homologous recombination [61,70,71]. The FISH analysis showed that these structural variations were inherited from a paternal balanced translocation. A terminal deletion within 8p is associated with hyperactivity, as well as with ID and impulsivity, whereas 4p terminal duplications are associated with microcephaly, motor retardation with seizures, and postnatal growth alterations [62–66]. Although translocation between chromosomes 4 and 8 is common, only a few cases of partial trisomy 4p and partial monosomy 8p have been reported [57–60,70]. The descriptions provided in the literature do not allow a typical phenotype of this type of dysmorphic syndrome to be assumed, because the distribution of dysmorphisms among patients is heterogeneous and the observed facial dysmorphisms are not specific.

Enrichment analysis confirmed that the gene list from the 4p16.3 region is enriched with genes associated with craniofacial and musculoskeletal abnormalities, which could explain the dysmorphic facial and growth features observed in the siblings evaluated here. Genes present in the 4p16.3 duplication and 8p23.3 deletion are involved in the cell cycle and cell differentiation, and some of these genes are likely to be more specifically related to the observed dysmorphic features in the siblings, such as *FGFR3* associated with chondrogenic and osteogenic events [72], and well known to be related to skeletal dysplasia [73]. Although the similarity of traits between the siblings could be explained by their unbalanced translocation, it is of note that cases in the literature sharing similar chromosomal abnormalities have not presented the same psychiatric symptoms.

In keeping with the hypotheses of network medicine proposed by Barabási et al. [53], the results from our network analysis of the siblings show that genes within the unbalanced translocation and the probable deleterious exome variants appeared to be connected in their biggest component. In addition, some genes that were disconnected only from the genes within the unbalanced translocation in our network analysis (S1 Fig) became connected when the exome results were included in the analysis. *MXD4*, *SH3BP2*, *ZFYVE28*, *LRPAP1* and *RGS12* appeared in both networks, whereas *LYAR* and *NOP14* became connected only in the network of the male sibling, *ADD1*, *ADRA2C*, *HTT*, and *CTBP1* being connected only in that of the female sibling.

According to Lim et al. [74], 5% of ASD cases can be affected by homozygous inherited rare variations, with loss of function, or hemizygous X-chromosome variations in males, and that deleterious mutations in X chromosome are more common in cases than in controls. In the present study, *FIGF* is the only gene that was connected in the biggest component of the network of the male sibling. In addition to being related to cell proliferation [74,75], probably contributing to the syndromic phenotype seen in our male patient, *FIGF* expression is modulated by activation of the Wnt signaling pathway [75] and is also related to dopaminergic neuron differentiation [76]. The Wnt pathway is one of the main pathways that have been associated with exome variants in ASD [77].

Pavlopoulos et al. [78] found that different types of biological networks can be modeled using graph theory, and that some of the nodes in a network may have topological properties that stand out in comparison with the others. The *CAD* gene, a variant of which was observed only in the male sibling evaluated here, is an important and highly connected broker gene. *CAD* encodes a protein associated with the enzymatic activities of the first three enzymes in the pyrimidine biosynthesis pathway and is regulated by the mitogen-activated protein kinase cascade [79]. In ASD patients, *CAD* has been found to be altered by 1 deleterious SNV and present in 4 CNVs [4]; it is also one of the genes identified by Samocha et al. [80] as being enriched with *de novo* loss-of-function mutations in ASD patients and not in individuals without ASD.

Exclusively in our female patient, a variant of the *ITGB4* gene was a broker in the network. *ITGB4* encodes an integrin-associated transmembrane glycoprotein receptor, expressed on epithelial cells, and in neural cells such as astrocytes, Schwann cells, neurons, and neural stem cells [81]. In the central nervous system, beta4 integrin is expressed primarily in arterioles. Although a lack of endothelial beta4 integrin has no effect on vascular development, its absence in the hypoxic central nervous system disrupts transforming growth factor-beta–mediated signaling and results in defective arteriolar remodeling [82].

Among the exclusive variant genes within the network of the female sibling evaluated in the present study, *SPTBN4* functions as a bridge. It encodes the myelinated neuron-rich cytoskeleton protein beta-spectrin [83], which is important to the clustering of voltage-gated sodium channel axon initial segments and the nodes of Ranvier [84], being required for *ANK3* function [85]. Although not a broker or a bridge, *ANK3* was also connected in the network of the female sibling. *ANK3* encodes the protein ankyrin-G, also located at the nodes of Ranvier and the axon initial segment, a protein responsible for the generation of action potentials [86]. *ANK3* has been associated with autism susceptibility [87], as well as with increased risk for and modulation of working memory deficits in bipolar disorder and schizophrenia [88–90].

Another important female exclusive variant gene, *RELN*, encodes reelin, an extracellular matrix protein that activates a signaling pathway for neuronal migration and synaptic plasticity during brain development, as well as appearing to be involved in schizophrenia, autism, bipolar disorder, and other neuropsychiatric disorders [91–95]. In addition, a recent genome-wide association study of schizophrenia revealed that the strongest association was with a marker within *RELN*, and only in women [96]. Furthermore, Goes et al. [91] found evidence that genetic variation in *RELN* is associated with susceptibility to bipolar disorder, particularly in females.

Some of the altered genes shared by the two siblings evaluated in the present study functioned as hubs in only one of the siblings, differentially contributing to the stability of the gene network and probably the phenotypes. For instance, *IDUA* (present in duplication 4p) and *NCKIPSD* (present in a shared nonsynonymous SNV) were bridge genes only in the network of the male sibling.

The only *de novo* exome variant, *TUBAL3* (found in the female sibling), was not connected in the network. According to Veltman & Brunner [97], approximately 74 novel SNVs arise per genome per generation. Given the fact that deleterious *de novo* variants increase disease risk, various computational methods have been proposed to predict impact and gene prioritization in human disease [98–100]. However, even though we could not exclude *de novo TUBAL3* variation from our analysis, the integrative approach we used showed that it is not important to the understanding of the phenotypic differences between the two siblings.

Adding exome genes to the network of each sibling showed that, as stated by Andrews et al. [30], the more connected the genes were, the more severe was the clinical presentation. The male sibling had a network that was more well connected, and, with the inclusion of the exome, more genes from the translocation were included. Accordingly, his CARS score was indicative of greater severity and genes with deleterious variations exclusive to his network are clearly related to ASD. The female had some psychiatric symptoms such as excess sociability, periods of sleep difficulties, and periods of talkativeness, a presentation similar to that of a mood disorder. Notably, the variants found only in the female sibling, although having been associated with autism, have also been shown to be strongly associated with other psychotic disorders, which probably explains her broader phenotype.

## Conclusions

One of the limitations of the current study is the sample size, which was small for an integrative analysis, given that we accessed only the exome data of the two affected siblings. Therefore, we chose to leave the inherited CNV out of the analysis and focus on the variations that were the most likely to have deleterious effects, such as *de novo* CNVs, as well as *de novo* and rare inherited nonsynonymous SNVs. Nevertheless, with this analysis of only one family, we were able to show the importance of exploring the genotype-phenotype variability beyond the effect of one SNV or CNV.

Our results show that the translocation shared between the siblings could explain their similar syndromic phenotype, such as their overgrowth, macrocephaly, and ID. However, our network analysis showed that exome data aggregate genes to those already connected from their shared CNV, which are important to the robustness of the network and possibly contribute to the understanding of the broader spectrum of psychiatric symptoms.

## Supporting Information

S1 Fig MLPA results. (A) female sibling, (B) male sibling, (C) father, and (D) mother. In A and B, the dot over the 1.3 line of the peak ratio value represents the 4p16.3 duplication, and the dot under the 0.7 line of the peak ratio value represents the 8p23.3 deletion.
(TIF)

S2 Fig STRING network of genes present in the chromosomal alteration der(8)t(4;8)(p16.3;p23.1) in the siblings. The biggest connected component is composed of 17 proteins with 20 interactions (i.e., 24.6%, 17 of the 69 brain-expressed genes), 6 connected components composed of 2 proteins with 1 interaction and 1 connected component with 3 proteins and 2 interactions, resulting in 15 proteins (i.e., 21.7%, 14 of the 69 brain-expressed genes) and 8 interactions.
(TIF)

S1 Table Summary of the medical history and clinical evaluation of the probands. (DOCX)

S2 Table List of *de novo* CNVs found with 180K aCGH in the siblings. (DOCX)

S3 Table Results of WebGestalt disease enrichment analysis with at least 4 genes from the 4p16.3 duplication gene list (69 brain-expressed genes) (DOCX)

S4 Table Summary of exome sequencing data quality of each member of the family. (DOCX)

S5 Table List of deleterious variants after filtering in the male and female sibling exome data. (XLSX)

S6 Table Gene list with centrality measures of the biggest connected component of the network of the male sibling. (XLSX)

S7 Table Gene list with centrality measures of the biggest connected component of the network of the female sibling. (XLSX)
