# Supplementary figures and images for: Integrative Variation Analysis Reveals that a Complex Genotype May Specify Phenotype in Siblings with Syndromic Autism Spectrum Disorder
Source: PLoS One. 2017 Jan 24;12(1):e0170386. doi: 10.1371/journal.pone.0170386 (PMC5261619; doi:10.1371/journal.pone.0170386)

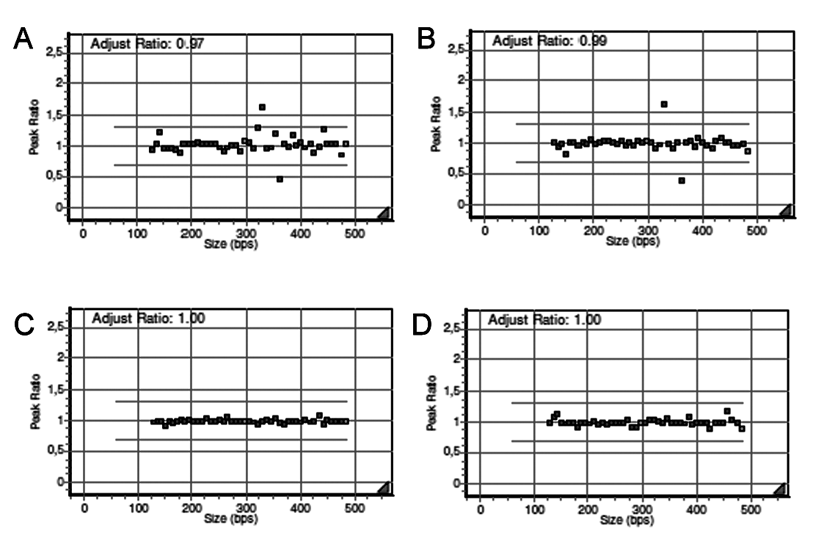

Supplement: S1 Fig — (A) female sibling, (B) male sibling, (C) father, and (D) mother. In A and B, the dot over the 1.3 line of the peak ratio value represents the 4p16.3 duplication, and the dot under the 0.7 line of the peak ratio value represents the 8p23.3 deletion. (TIF) [file pone.0170386.s001.tif]

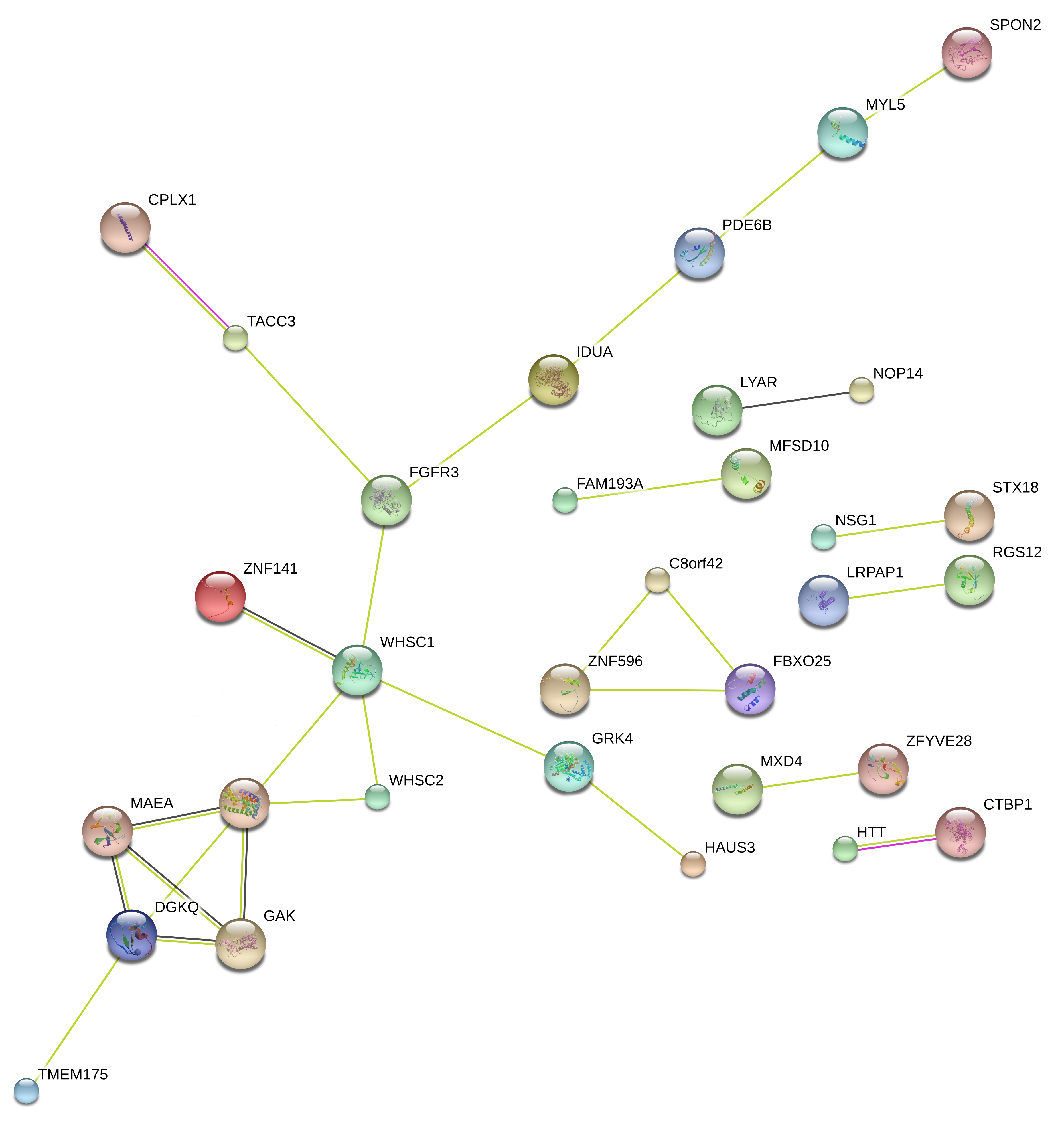

Supplement: S2 Fig — The biggest connected component is composed of 17 proteins with 20 interactions (i.e., 24.6%, 17 of the 69 brain-expressed genes), 6 connected components composed of 2 proteins with 1 interaction and 1 connected component with 3 proteins and 2 interactions, resulting in 15 proteins (i.e., 21.7%, 14 of the 69 brain-expressed genes) and 8 interactions. (TIF) [file pone.0170386.s002.tif]
