# Supplementary material for: Integrative Variation Analysis Reveals that a Complex Genotype May Specify Phenotype in Siblings with Syndromic Autism Spectrum Disorder
Source: PLoS One. 2017 Jan 24;12(1):e0170386. doi: 10.1371/journal.pone.0170386 (PMC5261619; doi:10.1371/journal.pone.0170386)
Supplement: S1 Table — (DOCX) [file pone.0170386.s003.docx]

**S1 Table. Summary of the medical history and clinical evaluation of the probands.**

|  | **Male sibling** | **Female sibling** |
| --- | --- | --- |
| **Age at evaluation** | 33 | 29 |
| **Psychiatric examination** | - Did not sustain eye contact or initiate interaction  - Agitation  - Unmotivated laughter; inappropriate social contact  - Understood simple commands  - Difficulty obeying some commands  - No imitation  - Non-verbal (guttural sounds and screams are present); absence of functional communication  - Presented syndromic features and probably ID | - Presented eye contact and initiated interaction in an inappropriate form, quite puerile but with no functionality  - Easy (motivated and unmotivated) laughter and collaborative  - Understood simple and (some) complex commands  - Used imitation  - Verbal, simple sentences with substitutions (mostly "r" for "l").  - Presented syndromic features and probably ID |
| **Neurological evaluation** | - Kyphoscoliosis  - All deep reflexes were symmetric but slightly diminished  -Discrete hypotonia  -Gait was symmetric with large steps; no alteration of gait stability | - Kyphosis  - Exhibited some difficulty standing on one foot  - Gait was symmetric with large steps; no alteration of gait stability |
