# Supplementary material for: Integrative Variation Analysis Reveals that a Complex Genotype May Specify Phenotype in Siblings with Syndromic Autism Spectrum Disorder
Source: PLoS One. 2017 Jan 24;12(1):e0170386. doi: 10.1371/journal.pone.0170386 (PMC5261619; doi:10.1371/journal.pone.0170386)
Supplement: S2 Table — (DOCX) [file pone.0170386.s004.docx]

**S2 Table. List of de novo CNVs found with 180K aCGH in the siblings**

| **Sibling** | **Chr** | **Cytoband** | **Start** | **Stop** | **Size** | **Type** | **Genic** | **Pathogenicity^2^** |
| --- | --- | --- | --- | --- | --- | --- | --- | --- |
| Female | chr2 | p11.2 - p11.1 | 89441848 | 91766271 | 2.324.423 | Dup | No | Benign CNV Region |
| Female | chr4 | p16.3 - p16.2 | 71552 | 4673343 | 4.601.791 | Dup^1^ | Yes | ***Pathogenic CNV Region*** |
| Female | chr4 | q13.2 | 69392545 | 69462438 | 69.893 | Dup | Yes | Benign/Likely Benign CNV Region |
| Female | chr8 | p23.3 | 176814 | 928886 | 752.072 | Del^1^ | Yes | ***Uncertain/Pathogenic CNV Region*** |
| Female | chr8 | q24.3 | 145181640 | 145244005 | 62.365 | Dup | Yes | Benign/Likely Benign CNV Region |
| Female | chr14 | q32.33 | 106803248 | 106931193 | 127.945 | Dup | No | Benign CNV Region |
| Female | chr15 | q11.1 - q11.2 | 20564608 | 22558756 | 1.994.148 | Del | Yes | Benign CNV Region |
| Female | chr15 | q14 | 34735949 | 34806953 | 71.004 | Del | No | Benign/Likely Benign CNV Region |
| Male | chr4 | p16.3 - p16.2 | 71552 | 4673343 | 4.601.791 | Dup^1^ | Yes | ***Pathogenic CNV Region*** |
| Male | chr5 | p15.33 | 723194 | 777000 | 53.806 | Del | No | Benign CNV Region |
| Male | chr8 | p23.3 | 176814 | 928886 | 752.072 | Del^1^ | Yes | ***Uncertain/Pathogenic CNV Region*** |
| Male | chr8 | q24.3 | 145181640 | 145258838 | 77.198 | Dup | Yes | Benign/Likely Benign CNV Region |
| ^1^ der(8)t(4;8)(p16.3;p23.1) | | | | | | | | |
| ^2^ According to CNVs of similar sizes and region reported at Clinvar (doi: 10.1093/nar/gkv1222) | | | | | | | | |
