# Supplementary material for: Integrative Variation Analysis Reveals that a Complex Genotype May Specify Phenotype in Siblings with Syndromic Autism Spectrum Disorder
Source: PLoS One. 2017 Jan 24;12(1):e0170386. doi: 10.1371/journal.pone.0170386 (PMC5261619; doi:10.1371/journal.pone.0170386)
Supplement: S3 Table — (DOCX) [file pone.0170386.s005.docx]

**S3 Table.** Results of WebGestalt disease enrichment analysis with at least 4 genes from the 4p16.3 duplication gene list (69 brain-expressed genes)

| **Disease** | **#Gene*** | **EntrezGene** | **Cat** | **Obs** | **Exp** | **Ratio** | ***p*-value**** | **Adjusted**  ***p*-value***** |
| --- | --- | --- | --- | --- | --- | --- | --- | --- |
| Huntington Disease | 7 | 317648; 2868; 118; 5158; 3064; 8603; 3425 | 66 | 7 | 0.11 | 66.29 | 1.42e-11 | 1.99e-10 |
| Chorea | 6 | 317648; 2868; 118; 5158; 3064; 8603 | 65 | 6 | 0.10 | 57.69 | 1.03e-09 | 7.21e-09 |
| Basal Ganglia Diseases | 5 | 2580; 3064; 8603; 84286; 1609 | 169 | 5 | 0.27 | 18.49 | 7.99e-06 | 3.73e-05 |
| Multiple Myeloma | 4 | 7468; 2261; 3083; 10460 | 132 | 4 | 0.21 | 18.94 | 6.21e-05 | 0.0001 |
| tardive dyskinesia | 5 | 2580; 3064; 8603; 84286; 1609 | 237 | 5 | 0.38 | 13.19 | 4.05e-05 | 0.0001 |
| Movement Disorders | 5 | 2580; 3064; 8603; 84286; 1609 | 237 | 5 | 0.38 | 13.19 | 4.05e-05 | 0.0001 |
| Syndrome | 7 | 7468; 2261; 285489; 3954; 7469; 3425; 57654 | 654 | 7 | 1.05 | 6.69 | 8.54e-05 | 0.0002 |
| Craniofacial Abnormalities | 5 | 7468; 2261; 3954; 6452; 53407 | 286 | 5 | 0.46 | 10.93 | 9.84e-05 | 0.0002 |
| Musculoskeletal Abnormalities | 5 | 7468; 2261; 3954; 6452; 53407 | 327 | 5 | 0.52 | 9.56 | 0.0002 | 0.0003 |
| Neurodegenerative Diseases | 5 | 4043; 2580; 3064; 1609; 57654 | 404 | 5 | 0.65 | 7.74 | 0.0005 | 0.0007 |
| Abnormalities, Multiple | 4 | 7468; 2261; 3954; 7469 | 340 | 4 | 0.54 | 7.35 | 0.0022 | 0.0028 |
| Vascular Diseases | 4 | 152; 7468; 2868; 118 | 357 | 4 | 0.57 | 7.00 | 0.0026 | 0.0030 |
| Congenital Abnormalities | 5 | 7468; 2261; 3954; 6452; 57654 | 643 | 5 | 1.03 | 4.86 | 0.0037 | 0.0040 |
| Cat: number of reference genes in the category; Obs: number of genes in the gene set and also in the category; Exp: expected number in the category; Ratio: ratio of enrichment ; *Showing results with at least 4 genes; **Hypergeometric statistical test; **Benjamini-Hochberg multiple test adjustment. | | | | | | | | |
|  | | | | | | | | |
