# Supplementary material for: Integrative Variation Analysis Reveals that a Complex Genotype May Specify Phenotype in Siblings with Syndromic Autism Spectrum Disorder
Source: PLoS One. 2017 Jan 24;12(1):e0170386. doi: 10.1371/journal.pone.0170386 (PMC5261619; doi:10.1371/journal.pone.0170386)
Supplement: S4 Table — (DOCX) [file pone.0170386.s006.docx]

**S4 Table.** Summary of exome sequencing data quality of each member of the family.

|  | Mother | Father | Male Sibling | Female Sibling |
| --- | --- | --- | --- | --- |
| Raw reads | 44,552,415 | 42,228,273 | 47,090,732 | 46,562,751 |
| Filtered reads | 32,079,570 | 30,839,118 | 33,716,494 | 33,386,916 |
| Mapped reads | 31,923,730 | 30,685,694 | 33,539,194 | 33,205,438 |
| Mapped base-pairs (Gb) | 3.2 | 3.1 | 3.4 | 3.3 |
| % reads aligned | 99.51 | 99.50 | 99.47 | 99.46 |
| % 20x coverage on target (per base) | 45.27 | 44.27 | 44.81 | 44.17 |
